# Altered ANG/TIE gene expression in advanced ovarian cancer is not accompanied by disproportionate body water accumulation relative to perioperative fluid balance

**DOI:** 10.1038/s41598-026-68951-3

**Published:** 2026-09-01

**Authors:** Sarah Reifenhaeuser, Katrin Stein, Stilla Frede, Eva K. Egger, Alexander Mustea, Mark Coburn, Sven Klaschik, Martin Soehle, Tobias Hilbert

**Affiliations:** 1 Department of Anesthesiology and Intensive Care Medicine, University Hospital Bonn, University of Bonn, Venusberg-Campus 1, 53127 Bonn, Germany; 2 Department of Obstetrics and Gynecological Oncology, University Hospital Bonn, University of Bonn, Venusberg-Campus 1, 53127 Bonn, Germany

**Keywords:** Endothelial permeability, Vascular leakage, Angiopoietin, Pulse pressure variation, Total body water, Extracellular water, Cancer, Diseases, Medical research, Oncology

## Abstract

Abdominal cytoreductive surgery due to advanced-stage ovarian cancer (OC) often requires extensive fluid administration to stabilize hemodynamic conditions. Recently, systemic changes in the expression profile of Angiopoietin (ANG)/TIE axis genes have been demonstrated in those patients, raising the question whether they exhibit an inherent clinical phenotype associated with vascular dysfunction and leakage that may explain the significant perioperative fluid demands. Abdominal muscle tissue gene expression analysis as well as pre- and postoperative whole-body bioelectrical impedance analysis (BIA) in patients with OC (*n* = 20) and in control subjects (*n* = 16) were performed, and Total Body Water (TBW), Extracellular Water (ECW) and the ECW/TBW ratio were calculated. Intraoperative management comprised advanced hemodynamic monitoring and goal-directed therapy. Data are given as median values with interquartile range (IQR). Postoperative fluid balance normalized to duration of surgery was greater in OC group (11.2 (IQR 9.8–13.2) vs. 8.3 mL/min (6.3–10.8) in controls, *p* = 0.01), as was the maximum body weight-adjusted norepinephrine administration rate (*p* < 0.001), whereas the intraoperative fluid administration rate did not differ between groups (*p* = 0.29). Gene expression analysis revealed significantly increased ANG-2/1 and ANG-2/TIE2 expression ratios in peripheral muscle tissue from OC patients (*p* < 0.01), while additional markers of angiogenesis, endothelial activation, adhesion, and vascular barrier integrity showed no significant intergroup differences. ANG-2/1 gene expression ratio was significantly associated with postoperative fluid balance in the whole cohort (*r* = 0.54, *p* < 0.001). There were no pre- or postoperative intergroup differences in TBW, ECW and in the ECW/TBW ratio. However, intraoperative percentage increase in BIA parameters was significantly more pronounced in the OC group, compared to controls (TBW: 12.3 (9.9–14.6) vs. 8.1% (5.2–9.0), *p* < 0.001; ECW: 21.7 (17.1–25.9) vs. 15.5% (11.0–18.1), *p* < 0.001; ECW/TBW: 8.7 (7.7–10.4) vs. 6.9% (5.1–8.5), *p* = 0.006). Importantly, the relationships between perioperative fluid balance and changes in TBW, ECW and ECW/TBW ratio did not differ between groups (group × fluid balance interactions: *p* = 0.614, *p* = 0.511, and *p* = 0.545, respectively). In contrast, these BIA-derived changes increased progressively with longer duration of surgery in OC patients but not in controls, resulting in significant group × duration of surgery interactions (*p* = 0.030, *p* = 0.018, and *p* = 0.037, respectively). Patients with advanced-stage OC exhibited altered ANG/TIE gene-expression profiles, greater vasopressor requirements, and a distinct time-dependent pattern of perioperative body-water accumulation during cytoreductive surgery. However, BIA did not demonstrate a disproportionate increase in body water relative to positive fluid balance compared with controls. Thus, the observed alterations in ANG/TIE gene expression were not accompanied by a detectable systemic vascular leakage phenotype using BIA.

**Supplementary Information:**

The online version contains supplementary material available at https://doi.org/10.1038/s41598-026-68951-3.

## Introduction

Solid tumor growth such as in ovarian cancer (OC) leads to local changes in vascular biology. This progress, known as *angiogenic switching*, is a hallmark of sustained tumor development and is triggered by progressive hypoxia in its central region. Changes in the expression profiles of genes related to Hypoxia-inducible Factor (HIF), such as Vascular Endothelial Growth Factor (VEGF) or those of the Angiopoietin (ANG)/TIE axis, induce the formation of new blood vessels from existing ones (angiogenesis), thus ensuring the supply necessary for further tumor growth, maintenance, and metastasis^1–6^.

OC is the leading cause of death among gynecological malignancies and remains a major contributor to cancer mortality in women worldwide^7^. Although curative therapeutic options are limited with ongoing disease progression, standard treatment includes initial surgical cytoreduction (debulking), followed by adjuvant chemotherapy and, in some cases, additional anti-angiogenic therapy and poly-ADP-ribose polymerase inhibitors even in advanced-stage disease^8^. Abdominal debulking surgery is among the most invasive procedures in gynecology and is associated with significant hemodynamic changes, often requiring the administration of large amounts of fluid to stabilize the patient’s condition^9^. Since extensive fluid resuscitation is associated with poor postoperative outcomes^10–12^, goal-directed fluid therapy (GDT) is recommended to avoid volume overload^13^. However, it has been shown that vascular function is impaired during major gyn-oncological abdominal surgery despite the use of GDT^14^, which in turn likewise accounts for poor outcomes^15,16^.

We recently demonstrated a change in the systemic vascular biology of patients with OC^17^. The expression of ANG/TIE axis genes significantly differs from that observed in healthy cancer-free control subjects. These changes not only occurred in the tumor itself and in tumor-associated peritoneal tissue, but also in samples from peripheral muscle tissue that is not affected by tumor growth or metastases. As these expression profiles induce a genotype associated with vascular leakage, it is reasonable to question whether patients scheduled for cytoreductive surgery for advanced-stage OC also exhibit an inherent clinical phenotype associated with leakage. This would explain the hemodynamic deterioration and fluid requirements frequently observed during and after surgery. Our approach included the prospective collection of clinical data and tissue gene expression analysis, as well as intraoperative and perioperative whole-body bioelectrical impedance analysis (BIA) in patients with OC and in control subjects.

## Materials and methods

### Study design, patient information

This observational study was conducted in accordance with the Declaration of Helsinki and received prior approval from the institutional review board (IRB) of the University of Bonn (protocol number 360/16, date of approval March 21, 2018; amendments approved March 12, 2019 and November 10, 2020). Patients scheduled for laparotomy due to either a benign gynecological diagnosis (e.g. myomectomy, hysterectomy; referred to as control group) or due to primary cytoreductive surgery because of advanced-stage ovarian cancer (OC group) were prospectively screened to participate in the study. Exclusion criteria were as follows: inability or refusal to provide written consent, patient age < 18 years, pregnancy, prior malignant disease other than OC and an implanted pacemaker. A retrospective registration of the study has been done (German Clinical Trials Register, No. DRKS00041326).

Typically, patients in the OC group received a thoracic epidural catheter for postoperative analgesia prior to induction of general anesthesia. In all patients, anesthesia was induced using remifentanil, propofol and rocuronium followed by endotracheal intubation. Anesthesia maintenance was performed either in form of balanced anesthesia using sevoflurane or in form of total intravenous anesthesia using propofol, both combined with continuous infusion of remifentanil. As part of standard perioperative management, patients in OC group were equipped with a central venous catheter, a radial arterial catheter, and a feeding tube. A urinary catheter was placed in all patients.

Intraoperative hemodynamic management included the administration of crystalloids at a basal rate (4 ml/kg*h) and, if necessary, additional volume boluses to maintain discontinuously assessed pulse pressure variation (PPV) < 15%. The target mean arterial pressure (MAP) of at least 65 mmHg was achieved with continuous norepinephrine infusion. Blood loss was compensated in accordance with current national transfusion guidelines using colloids, red blood cell and platelet concentrates and Fresh Frozen Plasma.

Once surgical procedures were complete and patients were in a stable respiratory and cardiovascular condition, anesthesia was terminated and patients were extubated. They were then transferred to the general ward or intermediate care unit for further postoperative care.

### Collection of tissue samples and assessment of gene expression

Surgery was performed as median laparotomy. Tissue samples from abdominal wall muscle (M. rectus abdominis) were obtained by the surgical team immediately after abdominal wall incision in both control and OC group. Samples were stored in cryotubes and snap-frozen in liquid nitrogen without intentional delay and subsequently kept at -80 °C until further processing. Peripheral rectus muscle tissue was chosen to investigate whether alterations of the ANG/TIE axis extend beyond the local tumor microenvironment. As single vessel biopsies are not routinely obtainable during surgery and would themselves be subject to local vascular pathology, rectus muscle provided a standardized, non-tumorous tissue source that was equally accessible in both study groups. Consequently, the observed differences are more likely to reflect systemic rather than local tumor-associated changes in vascular biology.

RNA was isolated using TRIzol™ reagent (Fisher Scientific, Schwerte, Germany) according to the manufacturer’s instructions. After lysing and homogenizing samples in TRIzol™ reagent using a stand grinding unit (Polytron-Kinematica, Lucerne, Switzerland), triple phase separation was initiated by adding chloroform. While the red lower phase contains proteins and the interphase contains DNA, the RNA is located in the upper aqueous phase and was transferred to a new tube. Further RNA precipitation was achieved by adding isopropanol, resulting in a white gel-like pellet. The precipitated RNA was then washed with ethanol, air-dried and dissolved in RNAse-free diethyl pyrocarbonate (DEPC) treated water.

RNA concentration was assessed spectrophotometrically (NanoDrop, Thermo Scientific, Waltham, MA, USA). 2 µg of RNA was reverse transcribed in cDNA using the High Capacity cDNA Reverse Transcription Kit (Applied Biosystems, Weiterstadt, Germany), then diluted in DEPC-treated water and stored at + 4 °C.

Gene expression analyses of ANG-1, ANG-2, TIE2, VEGF, E-selectin, P-selectin, ICAM-1, VCAM-1, Syndecan-1 and Thrombomodulin were performed by quantitative real-time PCR using Taqman™ Expression Assays and Taqman™ Gene Expression Master Mix on a ViiA7 device (all Applied Biosystems, Weiterstadt, Germany).

Results are given as relative quantification (RQ) with the target gene normalized to 18 S ribosomal RNA as housekeeping-gene, calculated as the fold change expression of the mean expression level of the respective control (delta-delta CT method). All personnel performing RNA extraction, reverse transcription and qPCR measurements were blinded to the clinical and perioperative data of the recruited patients.

### BIA assessment

BIA measures impedance and phase angle of a generated alternating current. By using phase-sensitive measurement technology, the impedance is differentiated into two components: resistance (corresponding to the resistance generated by the intra- and extracellular fluid), and reactance (generated by the function of the body’s cells as capacitors). Multifrequency analysis allows to distinguish between the intracellular and extracellular compartment: while low-frequency currents propagate exclusively extracellularly, higher-frequency currents overcome cell membranes and thus also propagate in the intracellular fluid^18^.

Disposable electrodes were attached to the back of the patient’s dominant hand and foot, and the above-described current field was generated using a BIA system (Nutriguard-MS, Data Input GmbH, Pöcking, Germany). During measurement, patients were in a horizontal supine position, with the arms not touching the trunk and thighs kept apart. Measurements were performed at 5, 50 and 100 kHz. Fluid distribution was then calculated by the device’s internal software routine using resistance, reactance and phase angle, together with additional data (gender, age, body height and weight). For the study, Total Body Water (TBW), Extracellular Water (ECW) as well as the ECW/TBW ratio were calculated. BIA assessment was performed preoperatively immediately before induction of anesthesia and postoperatively before termination of anesthesia in both groups.

### Statistical and bioinformatical analysis

Data were transferred into MS Excel (Microsoft Corp., Redmond, CA, USA). Statistical analysis and visualization were performed using GraphPad PRISM 8 (La Jolla, CA, USA). All data are presented as median values with interquartile range (IQR). Significance of intergroup differences was tested using the Mann-Whitney test. Significance of differences between paired samples from two different time points was tested using the Wilcoxon signed-rank test. Fisher’s exact test was used for dichotomous parameters. Associations between different parameters were assessed using the Spearman rank correlation coefficient. No formal correction for multiple testing was applied, as the correlation analyses were predefined exploratory analyses investigating biologically related hypotheses. P values < 0.05 were considered statistically significant.

The datasets generated and analyzed during the current study are available from the corresponding author on reasonable request.

## Results

### Patient population

Forty consecutive patients were prospectively recruited to participate in the study. 16 otherwise healthy patients scheduled for surgery for benign gynecologic disease were recruited to the control cohort, and 24 patients scheduled for primary cytoreductive surgery for advanced-stage OC were recruited to the respective case cohort. While in the majority (*n* = 20 patients), histopathological analysis revealed advanced-stage ovarian cancer, four patients were diagnosed with either a malignant Brenner tumor with partial squamous differentiation, a serous cystadenofibromatous borderline tumor of the adnexa (*n* = 2) or a granulosa cell tumor. Those four patients were therefore excluded from the final analysis (Supplementary Figure S1).

Overall median patient age was 60 years (IQR 47–70), while patients in the OC group were significantly older than those in the control group (69 (61–71) vs. 44 years (38–53), *p* < 0.001). Duration of surgery was significantly increased in OC group (231 (165–281) vs. 126 min (110–152), *p* < 0.001), therefore, intraoperative fluid intake was likewise increased (3,274 (2,449–3,837) vs. 1,600 mL (1,251–2,000), *p* < 0.001). Since intraoperative urine output showed no significant differences, also postoperative fluid balance was increased in OC group (2,673 (2,046 − 3,254) vs. 1,200 mL (845–1,450) in control group, *p* < 0.001). When normalized to duration of surgery, the fluid administration rate did not differ between groups (14.0 (13.0–15.8) vs. 13.0 mL/min (10.0–14.8), *p* = 0.29). Adjusted urine output was likewise comparable, whereas estimated blood loss per minute was higher in the control group (1.75 (1.01–2.20) vs. 1.08 mL/min (0.41–1.60), *p* = 0.03), leading to a higher positive fluid balance per minute in the OC group (11.2 (9.8–13.2) vs. 8.3 mL/min (6.3–10.8), *p* = 0.01). Moreover, the maximum body weight-adjusted norepinephrine administration rate for hemodynamic stabilization was significantly increased in OC patients (*p* < 0.001). No difference was found in norepinephrine administration between patients that received an epidural catheter and those who did not (*p* = 0.53).

Table 1 provides an overview of the basic patients’ characteristics and the procedural details.

**Table 1:** Patient and procedural details.

Parameter	Control	Ovarian cancer	*p* value
Patient details			
n	16	20	
Age (years)	44 (38–53)	69 (61–71)	***< 0.001***
Height (cm)	167 (159–170)	168 (165–172)	*0.43*
Weight (kg)	73 (66–84)	74 (61–91)	*0.82*
Body mass index (kg/m^2^)	26.5 (22.8–31.1)	24.5 (21.9–32.7)	*0.83*
Body surface area (m^2^)	1.8 (1.7–1.9)	1.8 (1.7–2.0)	*0.58*
Diagnosed with:			
Uterine myoma (n [%])	11 (69)	-	
Ovarian cyst / cystadenoma (n [%])	3 (19)	-	
Ovarian fibroma (n [%])	1 (6)	-	
Endometriosis (n [%])	1 (6)	-	
Procedural details			
Duration of surgery (min)	126 (110–152)	231 (165–281)	***< 0.001***
Intraop. fluid intake (mL)	1,600 (1,251–2,000)	3,274 (2,449–3,837)	***< 0.001***
Intraop. fluid intake (mL/min)	13.0 (10.0–14.8)	14.0 (13.0–15.8)	*0.29*
Postop. fluid balance (mL)	1,200 (845–1,450)	2,673 (2,046 − 3,254)	***< 0.001***
Postop. fluid balance (mL/min)	8.3 (6.3–10.8)	11.2 (9.8–13.2)	***0.01***
Intraop. urine output (mL)	200 (100–490)	350 (200–575)	*0.45*
Intraop. urine output (mL/min)	1.58 (0.93–3.92)	1.49 (0.89–2.07)	*0.36*
Estimated intraop. blood loss (mL)	200 (100–300)	225 (92.5–400)	*0.91*
Estimated intraop. blood loss (mL/min)	1.75 (1.01–2.20)	1.08 (0.41–1.60)	***0.03***
Patients with PRBC transfusion (n [%])	0 (0)	1 (5)	*> 0.99*
Of those: numbers of PRBC units transfused	–	2 (2–2)	
Numbers of Fresh Frozen Plasma units transfused	0 (0–0)	0 (0–2)	***0.02***
Numbers of platelet concentrate units transfused	0 (0–0)	0 (0–0)	*> 0.99*
Max. norepinephrine dosage rate (µg/kg*min)	0.00 (0.00–0.01)	0.11 (0.05–0.25)	***< 0.001***
Epidural catheter (n [%])	4 (25)	14 (70)	***0.02***

Data are given as percentage values or as median values with 25th and 75th percentile, respectively. Mann-Whitney U test for continuous data or Fisher’s exact test for dichotomous parameters were used. PRBC = Packed red blood cells.

Significant values are in bold.

### Gene expression analysis

**Fig. 1:**
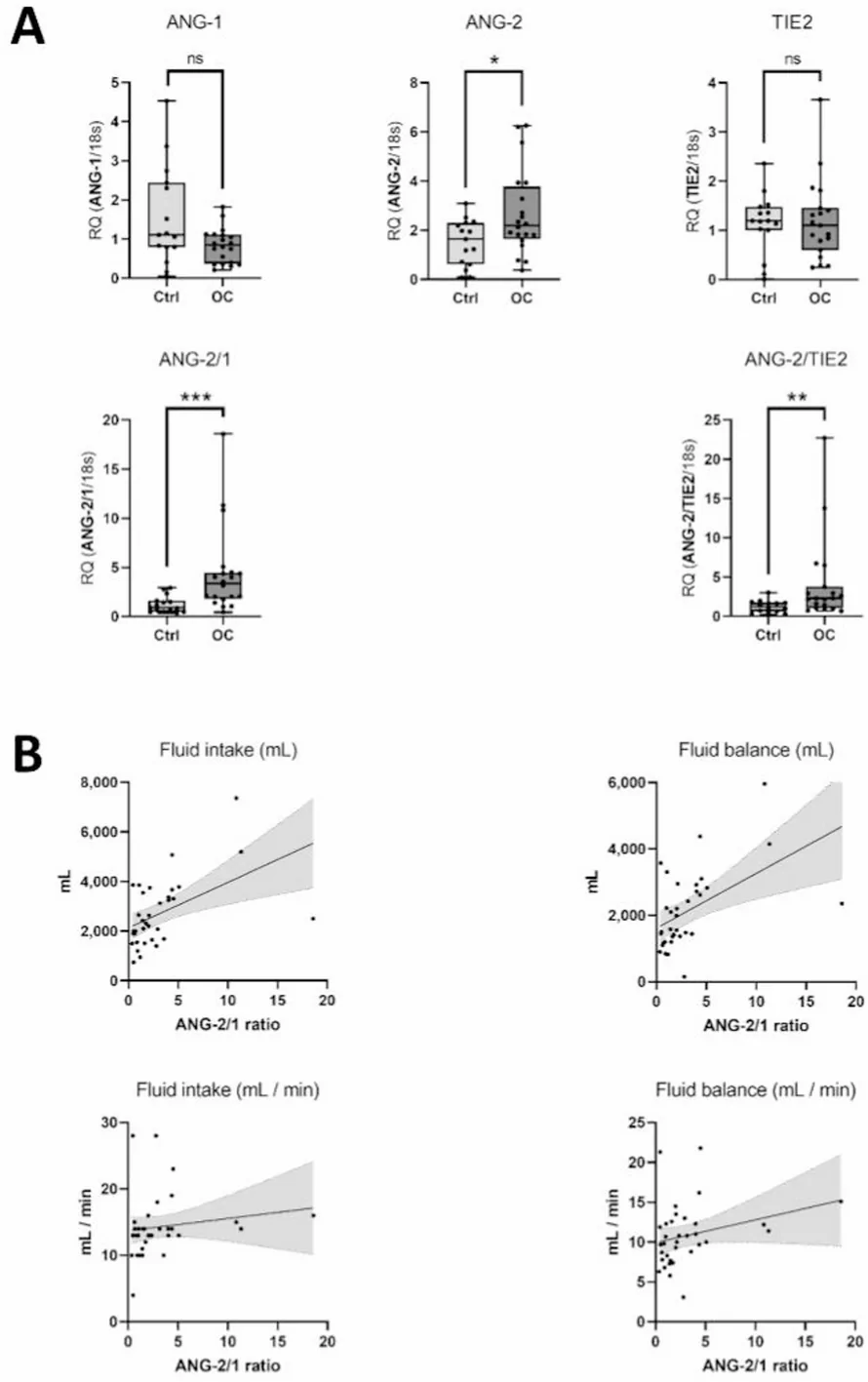
Abdominal wall muscle gene expression analysis. Tissue samples were collected from the abdominal wall musculature and gene expression analysis was performed using real-time PCR. (**A**) Figure shows expression of Angiopoietin-1 (ANG-1), ANG-2 and TIE2 in muscle tissue from ovarian cancer (OC) patients, compared to healthy controls (Ctrl). Data are given as median values with 25th and 75th percentile and range and were compared using Mann-Whitney U test. * *p* < 0.05, ** *p* < 0.01, *** *p* < 0.005. (**B**) Association of ANG-2/1 gene expression ratio with postoperative fluid intake and fluid balance. Spearman rank correlation coefficient. *n* = 16 for control group, *n* = 20 for OC group.

Tissue samples were collected from the peripheral muscle of the abdominal wall (M. rectus abdominis). The results of the gene expression analysis are shown in Fig. 1A and Supplementary Table S1. Genes from the ANG/TIE axis were significantly altered in OC patients. This seemed to induce a distinct systemic vascular leakage-related genotype, since ANG-2/1 as well as ANG-2/TIE2 ratio both were significantly increased, compared to the tumor-free control cohort. In the whole cohort, an increased ANG-2/1 gene expression ratio was significantly associated with increased postoperative fluid intake and fluid balance (*r* = 0.52, *p* = 0.001 and *r* = 0.54, *p* < 0.001, respectively) (Fig. 1B). This also applied when fluid intake and balance were normalized to the duration of surgery (*r* = 0.38, *p* = 0.02 and *r* = 0.39, *p* = 0.02, respectively). In contrast to a significant association between age and ANG-2/1 gene expression ratio in the whole cohort (*r* = 0.57, *p* = 0.0003), no such association was observed within the two subgroups.

Gene expression analyses of additional markers related to angiogenesis, endothelial activation, adhesion and vascular barrier integrity, including VEGF, E-selectin, P-selectin, ICAM-1, VCAM-1, Syndecan-1 and Thrombomodulin, revealed no significant differences between OC patients and controls (Supplementary Table S1).

### Whole-body bioelectrical impedance analysis

**Fig. 2:**
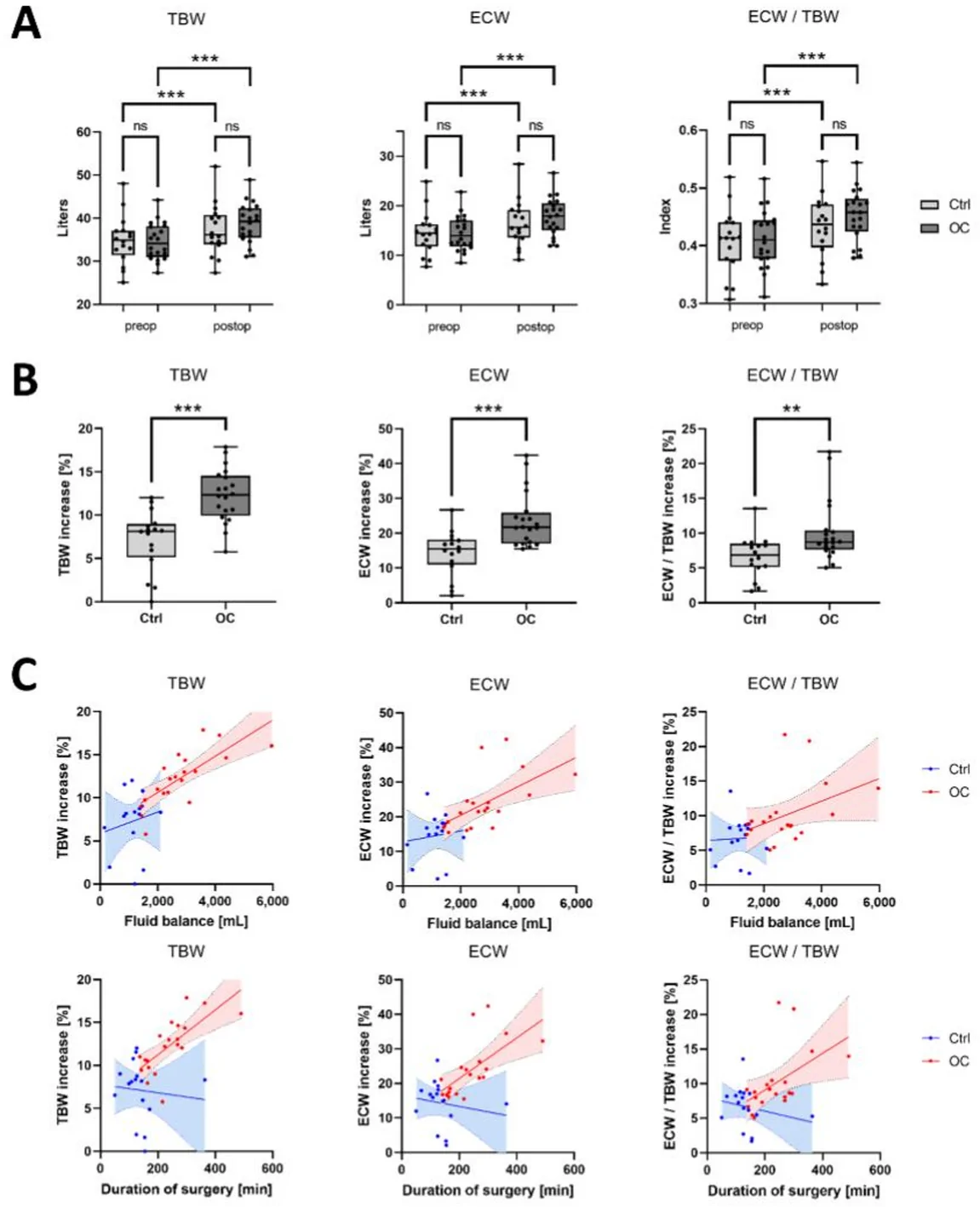
Whole-body bioelectrical impedance analysis. Bioelectrical impedance analysis was assessed before and immediately after surgery in all control and OC patients, and the Total Body Water (TBW), Extracellular Water (ECW) as well as the ECW/TBW ratio were calculated. (**A**) Figure shows pre- and postoperative TBW, ECW as well as ECW/TBW ratio in OC patients and controls. (**B**) Figure shows intraoperative percentage increase of TBW, ECW and ECW/TBW ratio in OC patients and controls. (**C**) Association between intraoperative percentage increase of TBW, ECW and ECW/TBW ratio and postoperative fluid balance in control (blue) and OC patients (red). Spearman rank correlation for whole cohort, dashed lines indicate the 95% confidence interval. (**A**, **B**) Data are given as median values with 25th and 75th percentile and range and were compared using Mann-Whitney U test or Wilcoxon signed-rank test, respectively. ** *p* < 0.01, *** *p* < 0.005. *n* = 16 for control group, *n* = 20 for OC group.

BIA was assessed in all control and OC patients before and immediately after surgery. Pre- as well as postoperatively, there were no intergroup differences in the BIA parameters TBW, ECW and in the ECW/TBW ratio (Fig. 2A). However, there was a significant increase in all three parameters from pre- to postoperative assessment in either group, suggesting intraoperative fluid accumulation in both control and OC patients. Pre- and postoperative values of TBW, ECW and ECW/TBW ratio are given in Supplementary Table S2.

Compared to the control group, percentage change in TBW, ECW and ECW/TBW ratio was significantly more pronounced in the OC group (TBW: 12.3 (9.9–14.6) vs. 8.1% (5.2–9.0), *p* < 0.001; ECW: 21.7 (17.1–25.9) vs. 15.5% (11.0–18.1), *p* < 0.001; ECW/TBW: 8.7 (7.7–10.4) vs. 6.9% (5.1–8.5), *p* = 0.006) (Fig. 2B). In the whole cohort, this percentage increase in TBW, ECW and the ECW/TBW ratio from pre- to postoperative assessment was significantly associated with the postoperative fluid balance (TBW: *r* = 0.77, *p* < 0.001; ECW: *r* = 0.64, *p* < 0.001; ECW/TBW ratio: *r* = 0.46, *p* = 0.006). Cases in OC group (with prolonged surgery) exhibited a more pronounced positive postoperative fluid balance, which was accompanied by a greater percentage increase in BIA parameters. To further investigate whether postoperative body water accumulation was primarily related to perioperative fluid administration or to the extent of surgical stress, linear regression analyses including study group, the explanatory variable, and their interaction were performed. When perioperative fluid balance was used as the explanatory variable, postoperative changes in TBW, ECW and ECW/TBW ratio increased with increasing fluid balance in both groups; however, the corresponding regression slopes did not differ significantly between ovarian cancer patients and controls (group × fluid balance interaction: TBW increase: *p* = 0.614; ECW increase: *p* = 0.511; ECW/TBW ratio increase: *p* = 0.545) (Fig. 2C). In contrast, regression analyses using duration of surgery as the explanatory variable demonstrated significantly different relationships between the study groups. Whereas no meaningful association between operative duration and postoperative changes in TBW, ECW, or ECW/TBW ratio was observed in controls, all three BIA-derived parameters increased progressively with longer duration of surgery in OC patients, resulting in significantly different regression slopes between groups (group × duration of surgery interaction: TBW increase: *p* = 0.0295; ECW increase: *p* = 0.0179; ECW/TBW ratio increase: *p* = 0.0373).

## Discussion

In this prospective observational cohort study, we found that patients with advanced-stage OC scheduled for cytoreductive surgery exhibited a distinct clinical and molecular phenotype, compared with benign controls. Clinically, the OC group received significantly greater amounts of intraoperative fluid during longer surgeries and required higher doses of norepinephrine to achieve hemodynamic stability. Whole‐body BIA revealed intraoperative fluid accumulation in both groups, as evidenced by increases in TBW, ECW, and ECW/TBW ratio. This increase was significantly greater in the OC group. Longer surgical duration was associated with progressively greater postoperative body water accumulation in OC patients, resulting in significant group differences in regression slopes. At the tissue level, gene expression analysis from rectus muscle tissue revealed significantly elevated ANG-2/1 and ANG-2/TIE2 ratios in OC patients compared with controls, suggesting a vascular leakage-associated genotype. Furthermore, the ANG-2/1 ratio was significantly correlated with an increased postoperative fluid balance.

Our data suggest that in advanced-stage OC, patients present with both molecular signs of vascular dysregulation and hemodynamic vulnerability during major debulking surgery.

There is an emerging body of evidence suggesting that the Angiopoietin/TIE2 axis plays a key role in tumor-associated vascular dysregulation, leakage and perioperative hemodynamic instability. In our study, patients undergoing debulking surgery received significantly more fluid intraoperatively in total than benign controls, which is consistent with recent findings^9,11,12,17^. The ANG/TIE2 pathway is well established as a regulator of vascular integrity. Under physiological conditions, ANG-1 promotes TIE2 activation, maintaining endothelial quiescence and barrier integrity, whereas ANG-2 antagonizes this effect, promoting endothelial destabilization and increased permeability^19–21^. In the tumor microenvironment, increased ANG-2 and decreased ANG-1 shift the balance towards a more leaky and unstable vasculature, which facilitates further tumor growth and metastasis. This has been described for multiple cancer entities, including OC^17,22,23^. Importantly, during major abdominal surgery for tumor masses, it has been shown that microvascular dysfunction progresses despite goal-directed fluid therapy (GDT)^14^. In line with that, we demonstrated that in gyn-oncological abdominal debulking surgery, a postoperative increase in ANG-2/1 ratio and consecutively impaired eNOS activity both correlate with fluid shifts and hemodynamic deterioration^24^.

Our own data extend this finding by showing that even peripheral (tumor-free) tissue exhibits this altered genotype (i.e., elevated ANG-2/1 as well as ANG-2/TIE2 ratio) in OC patients, suggesting a systemic vascular leakage‐related genotype that may predispose to fluid‐overload vulnerability and hemodynamic destabilization under surgical stress. We already previously demonstrated this dysregulation of the ANG/TIE axis in peripheral tissue samples of patients with advanced-stage OC^17^. Although the individual expression patterns of ANG-1, ANG-2, and TIE2 differed between the two studies, a significantly increased ANG-2/1 expression ratio was consistently observed. Moreover, re-analysis of the previous dataset revealed a significantly increased ANG-2/TIE2 ratio as well, confirming our current results. Therefore, the overall balance between stabilizing (ANG-1, TIE2) and disruptive (ANG-2) components of the ANG/TIE signaling axis, rather than isolated changes in individual genetic components (which may reflect cohort-specific characteristics such as age-dependent variability and biological heterogeneity), seems to represent the more robust and biologically relevant feature of systemic ANG/TIE dysregulation in advanced OC. With regard to genotype and phenotype, both studies revealed systemic ANG/TIE dysregulation being associated with intraoperative fluid intake. It should be noted, however, that although fluid balance was normalized to surgical duration, operation length represents only one surrogate of procedural complexity. Therefore, residual confounding arising from differences in surgical extent, tissue trauma, or inflammatory activation cannot be completely excluded.

Notably, the observed alterations in the ANG/TIE axis were not accompanied by significant changes in the expression of other genes involved in angiogenesis, endothelial activation, adhesion, or vascular barrier integrity, including VEGF, E-selectin, P-selectin, ICAM-1, VCAM-1, Syndecan-1 and Thrombomodulin. Although these negative findings should be interpreted cautiously (given the limited sample size), they may suggest that the observed transcriptional alterations are indicative of an endothelial activation profile that predominantly involves the ANG/TIE signaling axis.

In our study, BIA was significantly associated with fluid accumulation in both groups, with even more increased values in OC patients. BIA has been shown to be valid in assessing body fluid composition and intercompartmental shifts in open major abdominal surgery, and TBW and ECW significantly correlate with postoperative fluid balance^25^. Moreover, in critically ill as well as post-surgical patients, TBW and ECW have clearly been linked to increased vascular permeability and endothelial barrier disruption. The ECW/TBW ratio is therefore also referred to as *edema index*^26–28^. In our study, an increased ECW/TBW ratio in OC patients, together with altered angiopoietin expression, may, at first glance, suggest a specific mechanistic phenotype predisposing for increased vascular permeability. Interestingly, the relationship between perioperative fluid balance and postoperative changes in TBW, ECW, and ECW/TBW did not differ significantly between OC patients and controls, despite greater absolute postoperative fluid accumulation in the former. This suggests that perioperative fluid administration in OC patients in itself was not accompanied by a disproportionate increase in BIA-derived body water parameters per unit of positive fluid balance. Redistribution of crystalloid fluids from the intravascular to the interstitial compartment occurs rapidly even under physiological conditions, with only a minor fraction of approximately 20% remaining intravascular shortly after administration^29^. Consequently, the substantial interstitial fluid accumulation resulting from routine perioperative crystalloid administration may mask additional increases attributable to a disturbed endothelial barrier integrity, rendering them difficult to detect by BIA. This is in line with very recent findings from Feldheiser et al. who demonstrated (using venous congestion plethysmography) that fluid extravasation did not change throughout cytoreductive OC surgery despite a substantial positive fluid balance^30^.

In contrast, a significantly different relationship emerged when postoperative BIA changes were analyzed in relation to duration of surgery instead of perioperative fluid balance. Whereas no meaningful association between operative duration and changes in TBW, ECW or ECW/TBW ratio was observed in controls, all three parameters increased progressively with longer duration of surgery in OC patients, resulting in significantly different regression slopes between groups. As already mentioned above, operative duration is likely not only to independently reflect the extent of fluid administration, but is also a surrogate for the cumulative burden of surgical trauma, inflammatory activation, and endothelial stress^31^. In this context, prolonged cytoreductive surgery may progressively aggravate an underlying endothelial vulnerability^14,24^, potentially related to the observed preexisting ANG/TIE axis dysregulation, thereby facilitating interstitial fluid accumulation beyond that expected from fluid administration alone. Consequently, the rate of intraoperative fluid administration did not differ between groups, and the higher normalized positive fluid balance in the OC group occurred in the context of lower estimated blood loss per minute rather than a higher fluid administration rate. Therefore, the increased postoperative body water accumulation cannot simply be explained by more liberal intraoperative fluid therapy.

Nevertheless, these findings should be interpreted with caution. Duration of surgery differed substantially between groups, with OC procedures being considerably longer than control procedures. Therefore, the observed interaction should be regarded as hypothesis-generating and requires confirmation in larger studies including procedures with comparable operative duration. Moreover, in addition to vascular permeability, lymphatic drainage represents another determinant of tissue fluid homeostasis. Therefore, BIA reflects the net effect of transvascular fluid filtration and lymphatic clearance, in addition to endothelial barrier function. Since lymphatic function was not assessed in the present study, altered lymphatic drainage cannot be excluded as a contributor to perioperative fluid handling in OC patients.

Our study has some other limitations. First, a relatively modest sample size (20 °C patients, 16 controls), which may affect generalizability and statistical power. Second, the OC group was significantly older than the controls, which may confound vascular responsiveness or fluid handling. Age strongly affects vascular compliance, vasopressor requirement and fluid responsiveness and may therefore contribute to the observed hemodynamic differences, independent of cancer-related altered vascular biology. Third, we measured gene expression but did not assess protein levels; therefore, post-transcriptional effects could differ. 18 S rRNA was used as the reference gene throughout the study because of its established stable expression in human skeletal muscle tissue^32^. However, the use of a single reference gene represents a limitation, compared with current MIQE recommendations. Fourth, we used BIA to determine barrier dysfunction. While this method has been shown to be a useful non‐invasive indicator, it cannot definitively locate fluid extravasation or partition between interstitial and third‐space compartments without any complementary methods. The significance of lymphatic drainage has already been mentioned, but ascites volume, the drainage of which may directly affect intraoperative BIA values, has not been systematically assessed. Moreover, BIA was assessed during surgery but not in the postoperative period. Intergroup differences regarding barrier integrity may become apparent on the first postoperative day or later^27,33,34^. Finally, although we demonstrate association, this does not necessarily prove causality between elevated ANG-2/1 expression ratio and increased fluid requirement. Residual confounding is possible.

On the other hand, strengths of our study include its prospective design and the consideration of molecular, functional as well as clinical perioperative data.

## Conclusions

Patients with advanced-stage ovarian cancer exhibited altered ANG/TIE gene-expression profiles in peripheral tissue, greater vasopressor requirements, and a distinct time-dependent pattern of perioperative body-water accumulation during cytoreductive surgery. However, BIA did not demonstrate a disproportionate increase in body water relative to positive fluid balance compared with benign controls. Thus, despite molecular alterations in the ANG/TIE axis and differences in perioperative fluid handling, our findings provide no evidence for an inherently increased global vascular permeability in advanced ovarian cancer. Further studies incorporating direct functional measures of endothelial barrier integrity and vascular vulnerability are required to determine the functional relevance of the observed transcriptional alterations.

## Supplementary Information

Below is the link to the electronic supplementary material.


Supplementary Material 1



Supplementary Material 2



Supplementary Material 3



Supplementary Material 4


## Data Availability

The datasets used and/or analyzed during the current study are available from the corresponding author on reasonable request.

## Abbreviations

ADP: Adenosine Diphosphate
ANG: Angiopoietin
BIA: Bioelectrical Impedance Analysis
cDNA: Complementary Deoxyribonucleic Acid
DNA: Deoxyribonucleic Acid
CT: Cycle Threshold
DEPC: Diethyl Pyrocarbonate
ECW: Extracellular Water
eNOS: Endothelial Nitric Oxide Synthase
GDT: Goal-Directed (fluid) Therapy
HIF: Hypoxia-Inducible Factor
IQR: Interquartile Range
IRB: Institutional Review Board
MAP: Mean Arterial Pressure
NO: Nitric Oxide
OC: Ovarian Cancer
PCR: Polymerase Chain Reaction
PPV: Pulse Pressure Variation
RQ: Relative Quantification
RNA: Ribonucleic Acid
TBW: Total Body Water
TIE: Tyrosine Kinase Receptor
VEGF: Vascular Endothelial Growth Factor
