# Supplementary material for: Altered ANG/TIE gene expression in advanced ovarian cancer is not accompanied by disproportionate body water accumulation relative to perioperative fluid balance
Source: Sci Rep. 2026 Sep 1;16:27413. doi: 10.1038/s41598-026-68951-3 (PMC13534688; doi:10.1038/s41598-026-68951-3)
Supplement: Supplementary file 1 [file 41598_2026_68951_MOESM2_ESM.docx]

**Table S1: Gene expression analysis in abdominal wall muscle tissue**

| Parameter | Control | Ovarian cancer | *p* |
| --- | --- | --- | --- |
| **Vascular leakage-related genes:** |  |  |  |
| ANG-1 | 1.11 (0.80 - 2.44) | 0.85 (0.37 - 1.12) | *0.11* |
| ANG-2 | 1.64 (0.61 - 2.30) | 2.19 (1.64 - 3.77) | ***0.047*** |
| TIE2 | 1.19 (1.00 - 1.48) | 1.10 (0.60 - 1.46) | *0.75* |
| VEGF | 1.06 (0.80 - 1.34) | 1.17 (0.84 - 1.58) | *0.65* |
| ***Adhesion molecules:*** |  |  |  |
| ICAM-1 | 0.44 (0.30 - 4.34) | 0.73 (0.30 - 1.31) | *0.61* |
| VCAM-1 | 1.08 (0.76 - 2.38) | 1.15 (0.83 - 2.75) | *0.54* |
| E-selectin | 4.03 (0.17 - 51.23) | 1.90 (0.36 - 7.89) | *0.96* |
| P-selectin | 1.11 (0.74 - 1.76) | 1.93 (0.66 - 2.61) | *0.48* |
| **Others** |  |  |  |
| Syndecan-1 | 0.92 (0.61 - 2.00) | 1.69 (1.12 - 5.59) | *0.14* |
| Thrombomodulin | 0.87 (0.48 - 2.33) | 0.75 (0.57 - 1.26) | *0.82* |

Data are given in RQ (Gene of Interest/18S-RNA) as median values with 25th and 75th percentile and were compared to expression of respective genes in control group using Mann-Whitney U test. eNOS = Endothelial Nitric Oxide Synthase, iNOS = Inducible Nitric Oxide Synthase, ANG-1 = Angiopoietin-1, ANG-2 = Angiopoietin-2, TIE2 = Tyrosine Kinase with Immunoglobulin-like and EGF-like domains 2, VEGF = Vascular Endothelial Growth Factor, VEGFR1 = VEGF receptor 1, VEGFR2 = VEGF receptor 2, ANG-2/1 = ratio between ANG-2 and -1 expression, VE-Cadherin = Vascular Endothelial Cadherin
