# Supplementary material for: Altered ANG/TIE gene expression in advanced ovarian cancer is not accompanied by disproportionate body water accumulation relative to perioperative fluid balance
Source: Sci Rep. 2026 Sep 1;16:27413. doi: 10.1038/s41598-026-68951-3 (PMC13534688; doi:10.1038/s41598-026-68951-3)
Supplement: Supplementary file 2 [file 41598_2026_68951_MOESM3_ESM.docx]

**Table S2: Whole-body bioelectrical impedance analysis**

| Parameter | Control | Ovarian cancer | *p* |
| --- | --- | --- | --- |
| ***Preoperative:*** |  |  |  |
| Total Body Water (TBW; Liters) | 34.9 (31.4 - 37.1) | 34.1 (31.0 - 38.2) | *0.88* |
| Extracellular Water (ECW; Liters) | 14.5 (11.8 - 16.3) | 14.0 (11.8 - 17.1) | *0.89* |
| ECW / TBW | 0.41 (0.37 - 0.44) | 0.41 (0.38 - 0.44) | *0.89* |
| ***Postoperative:*** |  |  |  |
| TBW (Liters) | 36.2 (33.9 - 40.8) | 39.2 (35.5 - 42.3) | *0.29* |
| ECW (Liters) | 15.7 (13.6 - 19.1) | 18.0 (15.1 - 20.5) | *0.25* |
| ECW / TBW | 0.44 (0.40 - 0.47) | 0.46 (0.42 - 0.48) | *0.22* |
| TBW (%) | 8.1 (5.2 - 9.0) | 12.3 (9.9 - 14.6) | ***<0.001*** |
| ECW (%) | 15.5 (11.0 - 18.1) | 21.7 (17.1 - 25.9) | ***<0.001*** |
| ECW / TBW (%) | 6.9 (5.1 - 8.5) | 8.7 (7.7 - 10.4) | ***0.006*** |
| TBW (% per mL balance) | 0.006 (0.005 - 0.010) | 0.004 (0.004 - 0.006) | ***0.006*** |
| ECW (% per mL balance) | 0.014 (0.012 - 0.018) | 0.008 (0.007 - 0.012) | ***0.005*** |
| ECW / TBW (% per mL balance) | 0.006 (0.005 - 0.008) | 0.003 (0.002 - 0.005) | ***0.007*** |

Data are given as percentage values or as median values with 25th and 75th percentile, respectively. Mann-Whitney U test was used.
