# Supplementary material for: Altered ANG/TIE gene expression in advanced ovarian cancer is not accompanied by disproportionate body water accumulation relative to perioperative fluid balance
Source: Sci Rep. 2026 Sep 1;16:27413. doi: 10.1038/s41598-026-68951-3 (PMC13534688; doi:10.1038/s41598-026-68951-3)
Supplement: Supplementary file 3 [file 41598_2026_68951_MOESM4_ESM.docx]

STROBE Statement—checklist of items that should be included in reports of observational studies

|  | Item No. | Recommendation | Page  No. | Relevant text from manuscript |
| --- | --- | --- | --- | --- |
| **Title and abstract** | 1 | (*a*) Indicate the study’s design with a commonly used term in the title or the abstract | 2 | Abstract, Methods: “...pre- and postoperative whole-body bioelectrical impedance analysis (BIA) in patients with OC (n = 20) and in control subjects (n = 16) were performed...” The observational design is stated in the Methods section. |
|  |  | (*b*) Provide in the abstract an informative and balanced summary of what was done and what was found | 2 | Structured abstract reports background, methods, principal molecular/BIA/hemodynamic results, interaction analyses, and a cautious conclusion that BIA did not demonstrate disproportionate body-water increase relative to positive fluid balance. |
| Introduction | | | |  |
| Background/rationale | 2 | Explain the scientific background and rationale for the investigation being reported | 4 | Background describes tumor-associated angiogenesis/ANG-TIE biology, perioperative fluid requirements during ovarian-cancer cytoreduction, prior evidence of systemic ANG/TIE dysregulation, and the rationale for testing whether this is accompanied by a vascular-leakage phenotype. |
| Objectives | 3 | State specific objectives, including any prespecified hypotheses | 4 | “...it is reasonable to question whether patients scheduled for cytoreductive surgery for advanced-stage OC also exhibit an inherent clinical phenotype associated with leakage.” The study combines clinical data, tissue gene-expression analysis, and perioperative BIA to address this question. |
| Methods | | | |  |
| Study design | 4 | Present key elements of study design early in the paper | 5 | “This observational study...” Patients scheduled for laparotomy for benign gynecological disease (control group) or primary cytoreductive surgery for advanced-stage ovarian cancer (OC group) were prospectively screened. |
| Setting | 5 | Describe the setting, locations, and relevant dates, including periods of recruitment, exposure, follow-up, and data collection | 5–7 | Single-center study at the University of Bonn/University Hospital Bonn. Prospective perioperative data collection included tissue sampling at laparotomy and BIA immediately before induction and before termination of anesthesia. Recruitment dates are not reported in the manuscript. |
| Participants | 6 | (*a*) *Cohort study*—Give the eligibility criteria, and the sources and methods of selection of participants. Describe methods of follow-up  *Case-control study*—Give the eligibility criteria, and the sources and methods of case ascertainment and control selection. Give the rationale for the choice of cases and controls  *Cross-sectional study*—Give the eligibility criteria, and the sources and methods of selection of participants | 5, 8 | Eligibility/selection: patients scheduled for laparotomy for benign gynecological disease or primary cytoreductive surgery for advanced-stage OC were prospectively screened. Exclusion criteria: inability/refusal to consent, age <18 years, pregnancy, prior malignancy other than OC, and pacemaker. Final histopathology determined inclusion in the OC analysis. |
|  |  | (*b*) *Cohort study*—For matched studies, give matching criteria and number of exposed and unexposed  *Case-control study*—For matched studies, give matching criteria and the number of controls per case | N/A | Not a matched study; no matching criteria were used. |
| Variables | 7 | Clearly define all outcomes, exposures, predictors, potential confounders, and effect modifiers. Give diagnostic criteria, if applicable | 5–10 | Clinical/perioperative variables and treatment targets are defined in Methods (e.g., PPV <15%, MAP ≥65 mmHg, fluid/vasopressor management); gene-expression variables and BIA-derived TBW, ECW and ECW/TBW are defined in Methods. Group definition is based on benign gynecologic disease versus advanced-stage OC confirmed by histopathology. |
| Data sources/ measurement | 8* | For each variable of interest, give sources of data and details of methods of assessment (measurement). Describe comparability of assessment methods if there is more than one group | 5–7 | Tissue sampling/qPCR methods are described on pp. 5–6; BIA device, electrode placement, frequencies, patient positioning, calculation and timing are described on pp. 6–7. The same tissue-sampling and BIA procedures were used in both groups. |
| Bias | 9 | Describe any efforts to address potential sources of bias | 5–7, 14 | Standardized perioperative management and identical BIA/tissue assessment procedures were used. Personnel performing RNA extraction, reverse transcription and qPCR were blinded to clinical/perioperative data. Potential residual confounding (including age and procedural differences) is acknowledged in the limitations. |
| Study size | 10 | Explain how the study size was arrived at | 8 | Forty consecutive patients were prospectively recruited (16 controls; 24 suspected advanced-stage OC). No formal sample-size/power calculation or other prospective study-size rationale is reported. |

Continued on next page

| Quantitative variables | 11 | Explain how quantitative variables were handled in the analyses. If applicable, describe which groupings were chosen and why | 7–10 | Continuous variables are reported as medians with IQR. Fluid intake, urine output, blood loss and fluid balance were additionally normalized to duration of surgery; BIA changes were expressed as percentage changes. No categorization of continuous variables is reported. |
| --- | --- | --- | --- | --- |
| Statistical methods | 12 | (*a*) Describe all statistical methods, including those used to control for confounding | 7 | Mann–Whitney test for intergroup comparisons, Wilcoxon signed-rank test for paired measurements, Fisher’s exact test for dichotomous variables, Spearman rank correlations, and linear regression analyses are used. No confounder-adjusted multivariable model is reported. |
|  |  | (*b*) Describe any methods used to examine subgroups and interactions | 8–10 | Subgroup analysis: norepinephrine administration was compared within the OC group by epidural-catheter status. Interaction analyses tested group × fluid balance and group × duration of surgery for postoperative changes in TBW, ECW and ECW/TBW. |
|  |  | (*c*) Explain how missing data were addressed | Not reported | The manuscript does not describe a method for handling missing data. |
|  |  | (*d*) *Cohort study*—If applicable, explain how loss to follow-up was addressed  *Case-control study*—If applicable, explain how matching of cases and controls was addressed  *Cross-sectional study*—If applicable, describe analytical methods taking account of sampling strategy | N/A | No longitudinal follow-up/loss to follow-up and no matching were applicable to the reported perioperative cohort. |
|  |  | (*e*) Describe any sensitivity analyses | Not reported | No sensitivity analysis is described in the current manuscript. |
| Results | | | | |
| Participants | 13* | (a) Report numbers of individuals at each stage of study—eg numbers potentially eligible, examined for eligibility, confirmed eligible, included in the study, completing follow-up, and analysed | 8 | Forty consecutive patients were recruited: 16 benign controls and 24 patients scheduled for primary cytoreductive surgery for advanced-stage OC; 4 of the latter were excluded after final histopathology, leaving 20 OC patients and 16 controls for analysis. |
|  |  | (b) Give reasons for non-participation at each stage | 8 | Four patients were excluded after histopathology because final diagnoses were malignant Brenner tumor with partial squamous differentiation (n=1), serous cystadenofibromatous borderline tumor (n=2), or granulosa cell tumor (n=1). |
|  |  | (c) Consider use of a flow diagram | Supplementary Fig. S1 | A study design and patient flow diagram is provided as Supplementary Figure S1. |
| Descriptive data | 14* | (a) Give characteristics of study participants (eg demographic, clinical, social) and information on exposures and potential confounders | 8, 21 | Patient and procedural characteristics are summarized in Table 1, including age, height, weight, BMI, body surface area, diagnoses, duration of surgery, fluid variables, blood loss, transfusion, maximum norepinephrine rate, and epidural-catheter use. |
|  |  | (b) Indicate number of participants with missing data for each variable of interest | Not reported | Numbers of participants with missing data for each variable are not explicitly reported. |
|  |  | (c) *Cohort study*—Summarise follow-up time (eg, average and total amount) | N/A | The study assesses the perioperative period and does not report longitudinal follow-up time. |
| Outcome data | 15* | *Cohort study*—Report numbers of outcome events or summary measures over time | 8–10 | Summary measures are reported for perioperative fluid balance, vasopressor requirement, gene-expression parameters, and pre-/postoperative BIA-derived TBW, ECW and ECW/TBW. |
|  |  | *Case-control study—*Report numbers in each exposure category, or summary measures of exposure | N/A | The manuscript is reported as a prospective observational cohort rather than a case-control study. |
|  |  | *Cross-sectional study—*Report numbers of outcome events or summary measures | N/A | Not a cross-sectional study. |
| Main results | 16 | (*a*) Give unadjusted estimates and, if applicable, confounder-adjusted estimates and their precision (eg, 95% confidence interval). Make clear which confounders were adjusted for and why they were included | 8–10, 21 | Unadjusted group comparisons, medians/IQRs and p values are reported in Results/Table 1 and Supplementary Tables. Correlations and group × explanatory-variable interaction p values are reported. No confounder-adjusted multivariable estimates are presented. |
|  |  | (*b*) Report category boundaries when continuous variables were categorized | N/A | Continuous variables were not categorized into analysis categories. |
|  |  | (*c*) If relevant, consider translating estimates of relative risk into absolute risk for a meaningful time period | N/A | No relative-risk estimates are reported; translation to absolute risk is not applicable. |

Continued on next page

| Other analyses | 17 | Report other analyses done—eg analyses of subgroups and interactions, and sensitivity analyses | 8–10 | Additional analyses include the OC epidural-status subgroup comparison, age–ANG-2/1 correlations within the whole cohort/subgroups, and regression interaction analyses for group × fluid balance and group × duration of surgery. No sensitivity analysis is reported. |
| --- | --- | --- | --- | --- |
| Discussion | | | | |
| Key results | 18 | Summarise key results with reference to study objectives | 11–15 | Discussion summarizes the main molecular, hemodynamic and BIA findings in relation to the study question, including greater absolute body-water accumulation but similar relationships between fluid balance and BIA changes across groups. |
| Limitations | 19 | Discuss limitations of the study, taking into account sources of potential bias or imprecision. Discuss both direction and magnitude of any potential bias | 13–14 | Limitations include modest sample size/statistical power, major age difference, lack of protein-level assessment, use of a single reference gene, limitations of BIA for localizing extravasation/third-space fluid, unmeasured lymphatic function and ascites volume, absence of later postoperative BIA, residual confounding, and inability to infer causality. |
| Interpretation | 20 | Give a cautious overall interpretation of results considering objectives, limitations, multiplicity of analyses, results from similar studies, and other relevant evidence | 11–15 | Discussion interprets ANG/TIE findings cautiously in light of BIA results, prior literature, procedural differences and possible confounding. The surgery-duration interaction is explicitly described as hypothesis-generating. Conclusions state that BIA did not demonstrate disproportionate body-water increase relative to positive fluid balance. |
| Generalisability | 21 | Discuss the generalisability (external validity) of the study results | 14 | Generalisability is addressed indirectly: the modest sample size is stated to “affect generalizability and statistical power,” and confirmation in larger studies with procedures of comparable operative duration is recommended. |
| Other information | |  | | |
| Funding | 22 | Give the source of funding and the role of the funders for the present study and, if applicable, for the original study on which the present article is based | 17 | “The study was financially supported by the University of Bonn, University Hospital Bonn (Project No. O-417.0005).” The role of the funder is not explicitly stated. |

*Give information separately for cases and controls in case-control studies and, if applicable, for exposed and unexposed groups in cohort and cross-sectional studies.

**Note:** An Explanation and Elaboration article discusses each checklist item and gives methodological background and published examples of transparent reporting. The STROBE checklist is best used in conjunction with this article (freely available on the Web sites of PLoS Medicine at http://www.plosmedicine.org/, Annals of Internal Medicine at http://www.annals.org/, and Epidemiology at http://www.epidem.com/). Information on the STROBE Initiative is available at www.strobe-statement.org.
